# A Case of Hepatic Malignant Solitary Fibrous Tumor: A Case Report and Review of the Literature

**DOI:** 10.1155/2023/2271690

**Published:** 2023-02-09

**Authors:** Zhiyan Fu, Evita B. Henderson-Jackson, Barbara A. Centeno, Gregory Y. Lauwers, Mihaela Druta, Daniel A. Anaya, Yukihiro Nakanishi

**Affiliations:** ^1^Department of Pathology, Moffitt Cancer Center, Tampa, FL, USA; ^2^Department of Medical Oncology, Moffitt Cancer Center, Tampa, FL, USA; ^3^Department of Gastrointestinal Oncology, Moffitt Cancer Center, Tampa, FL, USA

## Abstract

A 73-year-old man with a history of atrial myxoma and basal cell carcinoma presented with unexplained fever. Contrast-enhanced CT abdomen showed a large left hepatic lobe mass with early enhancement and delayed venous washout, concerning for hepatocellular carcinoma. Fine needle aspiration showed numerous spindle cells with malignant nuclear features, suggestive of malignant spindle cell neoplasm. The patient underwent left hepatectomy. The surgical specimen showed a well-circumscribe solid mass (14.6 × 13.0 × 10.0 cm) with necrosis. Histopathological examination revealed a proliferation of spindle tumor cells with characteristic staghorn-shaped blood vessels, frequent mitoses, and necrosis. The tumor cells showed strong and diffuse expression of CD34 and STAT6, confirming the diagnosis of malignant solitary fibrous tumor. Solitary fibrous tumor is a rare fibroblastic tumor characterized by a staghorn vasculature and NAB2-STAT6 gene rearrangement. Solitary fibrous tumor of the liver is a rare occurrence. Although most solitary fibrous tumors behave in a benign fashion, solitary fibrous tumors might act aggressively. This case is unique in that it demonstrates an excellent correlation between radiologic, macroscopic, and microscopic features which can contribute to the improvement of radiologic and pathologic diagnostic accuracy.

## 1. Introduction

Solitary fibrous tumor (SFT) is a rare fibroblastic tumor characterized by a staghorn vasculature and NAB2-STAT6 gene rearrangement [1, 2]. The tumor cells were regarded as derived from pericytes, thereby the tumor was historically referred to as hemangiopericytoma. Although the etiology and the line of differentiation remain unclear, fibroblastic/myofibroblastic origin has been supported. SFT is ubiquitous in soft tissues and occurs in any anatomic site, although it is most commonly seen in the pleura [2]. SFTs of the liver are rare with thus far 84 reports [3]. The average age of patients is 57 years (range 16–87) with a female/male ratio of 1.4 : 1.0 [3]. There have been 26 reported cases of malignant hepatic SFTs [3–26] (Table 1). Herein, we report a case of a 73-year-old man with malignant hepatic SFT. Although malignant hepatic SFT is extremely rare, SFT should be included in the differential diagnosis of a single large hepatic mass. Radiological findings often mimic those of hepatocellular carcinomas.

## 2. Case Presentation

**Figure 1:**
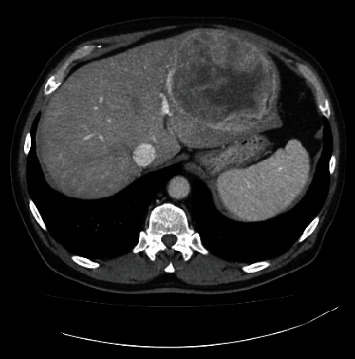
Contrast-enhanced abdominal computed tomography (CT) showed a large well-circumscribed mass in the lateral segment of the left hepatic lobe with early arterial phase enhancement.

**Figure 2:**
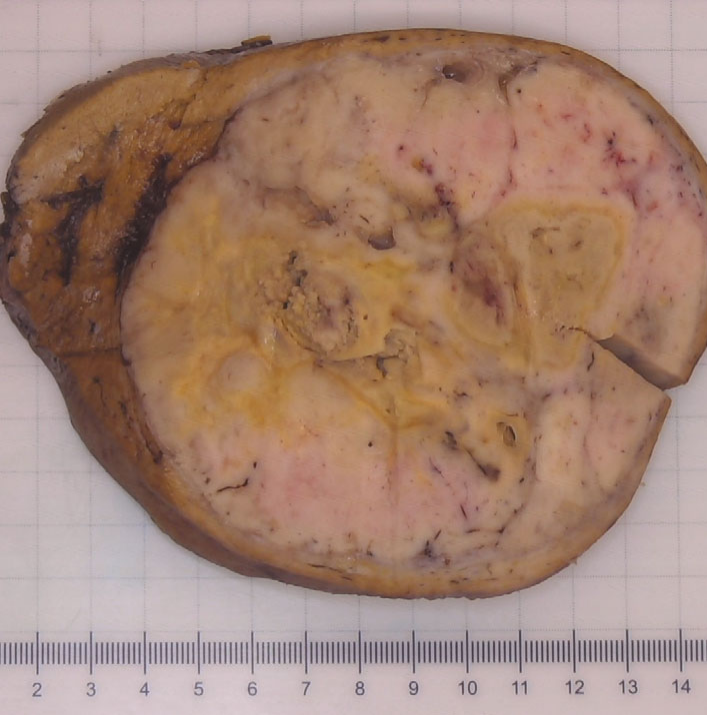
The surgical specimen showed a well-circumscribe solid mass (14.6 × 13.0 × 10.0 cm) with central necrosis.

**Figure 3:**
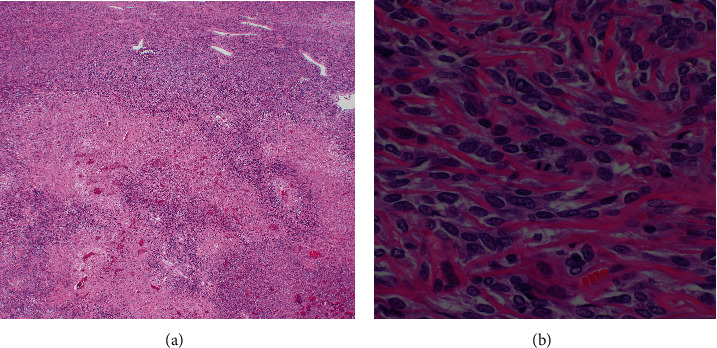
(a, b) The tumor was composed of haphazardly arranged spindle to ovoid cells with indistinct, pale cytoplasm, dense hyalinized collagenous stroma, frequent mitoses, necrosis, and characteristic staghorn-shaped blood vessels ((a) (40x) and (b) (400x)).

**Figure 4:**
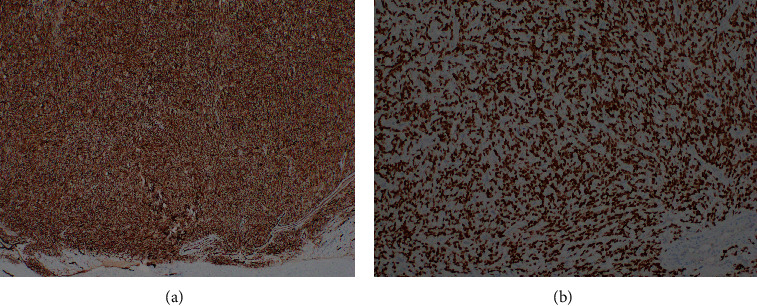
(a, b) The tumor cells showed strong and diffuse expression of CD34 ((a) 100x) and STAT6 ((b) 100x).

A 73-year-old man with a history of atrial myxoma and basal cell carcinoma presented with worsening lower extremity edema and unexplained fever. He initially presented to his primary care physician and cardiologist with these symptoms for a presumed cardiac etiology. An echocardiogram showed no signs of heart failure. Laboratory test results were unremarkable except for leukocytosis. During the work-up, a contrast-enhanced abdominal computed tomography (CT) showed a large left hepatic lobe mass with heterogenous enhancement in the periphery of the mass during the arterial phase. The enhancement became more pronounced in the areas surrounding necrotic changes during the portal phase (Figure 1) and eventually progressed to delayed venous phase washout. These radiologic findings suggested a high-grade hepatocellular carcinoma. No hepatic mass was seen in an abdominal CT in 2018. The patient was referred to our facility for further evaluation. The patient denies a history of hepatitis and alcohol abuse. Fine needle aspiration showed numerous spindle cells with malignant nuclear features, suggestive of malignant spindle cell neoplasm including sarcomatoid hepatocellular carcinoma. The patient with a tentative diagnosis of hepatocellular carcinoma underwent left hepatectomy. The surgical specimen showed a well-circumscribed yellowish-white solid mass (14.6 × 13.0 × 10.0 cm) with central necrosis (Figure 2). The mass demonstrated well-defined pushing borders with negative surgical margin. The background liver showed no significant fibrotic changes. Histopathological examination of the surgical specimen revealed a proliferation of spindle tumor cells with intercellular collagen bands and characteristic thin-walled, dilated, and branching staghorn-shaped blood vessels. Geographic necrosis, high cellularity, and frequent mitoses (more than four mitoses per 10 high power fields) are also noted (Figures 3(a) and 3(b)). No dedifferentiated component was identified. The tumor cells showed strong and diffuse expression of CD34 (Figure 4(a)) and STAT6 (Figure 4(b)), confirming the diagnosis of malignant solitary fibrous tumor (SFT). The patient is alive and disease-free at 13 months after hepatectomy.

## 3. Discussion

SFT is histopathologically characterized by a proliferation of fibroblastic spindle cells arranged in a patternless pattern within wire-like collagen and associated with a staghorn-like vasculatures. Other rare histopathologic features include myxoid change, mature adipose tissue, floret-like giant cells, and dedifferentiation. Dedifferentiated SFT represents the most aggressive SFT subtype. Recent studies using whole-exome sequencing and integrative sequencing demonstrated a fusion gene of juxtaposed NGFI-A-binding protein 2 (NAB2) and signal transducer and activator of transcription 6 (STAT6) in SFTs. STAT6 plays an important role as a potential key protein in the SFT pathogenesis. Therefore, STAT6 has been found to be a sensitive and specific marker for SFT and distinguishes SFT from its mimickers [2]. Other unspecific supportive immunostaining markers for SFT diagnosis include CD34, BCL-2, and CD99 [2]. Given the pathologic finding of a solitary large hepatic tumor along with the clinical finding of no metastasis, recurrence, or potential primary tumor in an extrahepatic organ at 13 months after hepatectomy, the overall findings of this case are consistent with a primary high-grade solitary fibrous tumor of the liver.

Prognostication of SFT is well known to be difficult [1]. Although the majority of tumors behave in a benign fashion, SFTs act aggressively, resulting in local recurrence and distant metastasis [1–26]. Although numerous studies have reported risk stratification models, it has been challenging to determine which clinicopathologic characteristics predict aggressive behavior. The most widely used risk stratification model classifies SFTs into low risk, intermediate risk, and high risk based on the total scores of clinicopathologic factors such as patient age (scored as 0 if <55 years and 1 if ≥55 years), mitotic activity (scored as 0 if <1 mitotic figure/10 HPF, 1 if 1–3 mitotic figures/10 HPF, or 2 if ≥4/10 HPF), and tumor size (scored as 0 if <5 cm, 1 if 5 to <10 cm, 2 if 10 to <15 cm, or 3 if ≥15 cm). Total scores were summed, and scores of 0–2 were considered as low risk, 3–4 as intermediate risk, and 5–6 as high risk [2]. Our case showed aggressive histologic features such as geographic necrosis, high cellularity, and frequent mitoses in addition to the patient's elderly age and large tumor size, consistent with high risk/malignant SFT.

Surgery is the mainstay of localized SFT treatment [2]. Complete surgical resection entails good prognosis. Although chemotherapy has been used in patients with advanced or metastatic SFTs, no clinical trials addressing the efficacy of chemotherapy have been reported [2]. The identification of the dysfunction in the transcript NAB2-STAT6 might lead to a development of a new therapeutic target such as EGR1 (early growth response 1), since the replacement of the repressor domain of NAB2 with the activation domain from STAT6 creates a potent transcriptional activator of EGR1 and constitutive activation of EGR-mediated transcription [2].

In summary, SFT should be included in the differential diagnosis of a single large hepatic mass with radiological findings mimicking hepatocellular carcinomas.

## Figures and Tables

**Table 1:** Summary of reported malignant hepatic solitary fibrous tumors.

Case #	Age/sex	Location in the liver	Tumor size (cm)	Management	Follow-up (months)	Outcome	Refs author/year
1	25/F	Right lobe and medial segment of the left lobe	32	Trisegmentectomy	6	Vertebrate metastasis	Yilmaz et al. 2000 [4]
2	71/F	Right lobe	14-17	Surgical resection	Not reported	Not reported	Fuksbrumer et al. 2000 [5]
3	74/M	Left lobe	24	Left hepatectomy	12	Disease-free	Terkivatan et al. 2006 [6]
4	61/F	Right lobe	21	Right trisegmentectomy	34	Disease-free	Nath et al. 2006 [7]
5	70/M	Right lobe	28	Segmental hepatectomy	12	Pulmonary metastasis	Chan et al. 2007 [8]
6	38/F	Left lobe	4	Surgical resection	11	Patient died with multiple liver and bone metastases	Seki et al. 2008 [9]
7	54/M	Right lobe	17	Surgical resection	96	Patient died with brain and bone metastases	Brochard et al. 2010 [10]
8	24/F	Right lobe	30	Transarterial chemoembolization and surgical resection	16	Patient died with skull base and bone metastases	Peng et al. 2011 [11]
9	66/F	Right lobe	14	Enucleation	30	Disease-free	Belga et al. 2012 [12]
10	62/F	Left lobe	20	Left hemihepatectomy	Not reported	Not reported	Jakob et al. 2013 [13]
11	78/M	Left lobe	17	Left hemihepatectomy	Not reported	Disease-free	Vythianathan et al. 2013 [14]
12	49/M	Not reported (multiple lesions)	7.6	Not reported	Not reported	Not reported	Song et al. 2014 [15]
13	74/F	Right lobe	24	Surgical resection	13	Pulmonary, mesenteric, omental, and abdominal wall metastases at 9 months after the surgery and died 4 months later	Maccio et al. 2015 [16]
14	80/F	Right lobe	19	Palliative chemotherapy	5	Patient died with pulmonary metastasis	Maccio et al. 2015 [16]
15	65/M	Right lobe	3	Chemotherapy	5	Patient died with pulmonary metastasis	Maccio et al. 2015 [16]
16	65/M	Left lobe	18	Left hemihepatectomy	16	Disease-free	Silvanto et al. 2015 [17]
17	55/F	Left lobe	15.5	Left extended hepatectomy	60	Local recurrence	Du et al. 2015 [18]
18	52/F	Right lobe	12	Surgical resection	37	Local recurrence	Feng et al. 2015 [19]
19	61/M	Right lobe	11.5	Subsegmental resection	72	Pleural metastasis	Chen et al. 2017 [3]
20	68/F	Right lobe	13.5	Right hepatectomy	37	Pulmonary metastasis	Esteves et al. 2017 [20]
21	78/F	Left lobe	18.5	Surgical resection	16	Patient died with recurrence	Barbosa et al. 2018 [21]
22	49/F	Right lobe	14	Central bisegmentectomy	12	Disease-free	Yugawa et al. 2019 [22]
23	17/F	Left lobe	21	Surgical resection	14	Disease-free	Shu et al. 2019 [23]
24	69/F	Right lobe	14	Chemotherapy	12	Patient died with pulmonary and subcutaneous metastases	Kim et al. 2019 [24]
25	69/M	Left lobe	10	Surgical resection	15	Pulmonary metastasis	Hoseini et al. 2020 [25]
26	47/M	Right lobe	30	Right hepatectomy	28	Disease-free	Yazdi et al. 2020 [26]
Present case	73/M	Left lobe	14.6	Left hepatectomy	13	Disease-free	

## Data Availability

Data including reports, patient details, and consent is stored on a secure drive accessible to the corresponding author.
